# The persuasive power of robot touch. Behavioral and evaluative consequences of non-functional touch from a robot

**DOI:** 10.1371/journal.pone.0249554

**Published:** 2021-05-05

**Authors:** Laura Hoffmann, Nicole C. Krämer

**Affiliations:** 1 Human-Centered Design of Socio-Digital Systems, Ruhr University Bochum, Bochum, Germany; 2 Social Psychology, Media and Communication, University of Duisburg-Essen, Duisburg, Germany; Nanyang Technological University, SINGAPORE

## Abstract

The unique physical embodiment of robots enables physical contact between machines and humans. Since interpersonal touch research has demonstrated that touch has several positive behavioral (e.g., reduced stress, better immune functioning) as well as evaluative consequences (e.g., better evaluation of the initiator of touch), the question arises whether touch from a humanoid robot, the body of which is somewhat similar to that of a human, can evoke similar effects. To answer this question, we conducted a between-subjects experiment in the laboratory with *n* = 48 students who encountered a humanoid robot (Softbank Robotics’ NAO) that either did or did not touch their hand in a non-functional manner during a counseling conversation. The analyses of participants’ behavior revealed that they mostly reacted by smiling and laughing. Furthermore, participants who were touched by the robot complied significantly more frequently with a request posed by the robot during conversation, and reported better feelings compared to those who were not touched. However, there were no effects of robot touch on subjective evaluations of the robot or on the interaction experience.

## Introduction

When robots increasingly enter our physical world in the (near) future, we may easily come into physical contact with them. This may occur accidentally, e.g., if a robotic vacuum cleaner lightly touches our feet, or purposely, e.g., if parts of the robot’s body need to be touched to execute a function or we simply want to touch the robot’s surface out of curiosity. At first glance, these examples might remind us of typical (physical) contact that we have with inanimate objects in our daily lives. However, when we consider that robots can have a human-like appearance and behavior, the question arises whether the experience of touch between humans and robots might have a different quality than mere touch with inanimate objects.

Indeed, research in the realm of human–robot interactions revealed that individuals who had tactile interactions with robots that had pet-like shapes showed improved vital signs such as lowered pain [1], anxiety [2], and blood pressure [3]. These findings resemble effects observed from therapies with living animals. Moreover, other studies revealed that touch from a robot that possesses body parts that allow for touch, e.g., arms, hands or grippers, evokes similar reactions to interpersonal touch. For instance, touch from a robot was found to result in increased physiological arousal [4,5], heightened motivation [6,7], and reduced feelings of unfairness [8]. However, recent work by Willemse, Toet and van Erp [9] did not observe any effect of a robot’s touch either on physiological arousal or on the evaluation of the robot.

Taken together, these findings suggest that tactile interactions with robots can, but do not necessarily, have noticeable effects on humans. Hence, more systematic research is needed to derive conclusions about the conditions that foster meaningful effects of robot touch. The present research therefore builds on previous findings from interpersonal as well as robot touch research and concentrates on the impact of touch from a humanoid robot (here: Softbank Robotic’s NAO) directed to human students. Furthermore, we address non-functional touch which is not connected to an instrumental goal (e.g., washing) and might thus be interpreted as an affective gesture. The main aims of the present work are as follows: 1) to implement non-functional robot-initiated touch without a prior need to touch the robot in advance (cf. [6,7]) in a controlled laboratory experiment, 2) to test hypotheses and application of measures derived from interpersonal touch research, 3) to investigate the immediate reactions to non-functional robot-initiated touch during a casual human–robot counseling interaction, and 4) to conduct a qualitative analysis of the meaning that humans assign to non-functional touch from a robot.

### Equating interpersonal touch with human–robot touch

The approach of using knowledge from the interpersonal literature and transferring it to humans’ interactions with technology is not new, and has frequently been applied in the realm of media equation research established by Clifford Nass and colleagues, e.g., [10,11]. The authors claim that humans’ interactions with technology are “fundamentally social” [12], which results in reactions to technological stimuli that resemble reactions to humans, e.g., answering politely if a computer or an embodied virtual agent asks a question about its own performance [13,14]. Additionally, these reactions are assumed to occur mindlessly, and indeed—on a conscious level—they are negated by those who show such reactions because they are regarded as inappropriate [11].

The direct transfer from interpersonal research findings to human–robot/ human–technology interaction is not free of criticism. In particular, the lack of comparison to a human control condition, as well as the existence of findings that showed differences in the treatment of humans and technologies, have been discussed (e.g., [15]). Nevertheless, such criticism does not diminish the usefulness of the approach for exploring a new research field such as human–robot interactions. In addition, findings in the realm of robot therapy support the transferability of findings from interactions with pets to robots (e.g., [16]). Although, touch between humans and animals is obviously different from interpersonal touch, we assume that building on existing interpersonal knowledge provides a fruitful starting point to investigate the implications of touch in human–robot encounters, although we neither suggest nor plan to test whether robot touch can be equated with interpersonal touch.

### Interpersonal touch research

Interpersonal touch generally means physical contact between two or more human individuals. In contrast to haptics research which focusses on the sense of touch as a skin sensation [17], interpersonal touch research focusses on effects that emerge as a result of touching or being touched by another individual. In this realm, touch has a rather communicative and relational function, and variables that are frequently investigated are in consequence relational closeness [18,19], prosocial behavior [20], health, and well-being [21–23].

A large body of research has investigated the meaning of touch and its impact on the recipient and initiator of touch (mostly in dyads). However, controlled analyses of interpersonal touch are no trivial undertaking: Natural observations are mostly restricted to touch in public settings (e.g., daily interactions [24]), and experimental manipulations are easily affected by confounding behaviors (e.g., gaze or speech that accompany experimentally induced touch [25]). For this reason, so far, interpersonal touch research has partly relied on the controlled observation of static situations captured in photographs (e.g., [26,27]), or on experimental studies in the field, where less intimate forms of touch such as a touch to the shoulder were introduced to investigate the impact of touch on, for example, compliance (e.g., [28–34]).

#### Meanings and positive effects of interpersonal touch

Interpersonal touch is a meaningful nonverbal communication channel because it is able to communicate distinct emotions [35] and relational messages [18,26]. This should be taken into account when investigating touch between humans and robots, since it has already been demonstrated that humans attribute meaning to the nonverbal behavior of a robot [36] and are able to communicate distinct emotions to robots via touch [37]. In the interpersonal context, Burgoon [26] demonstrated that different forms of touch have different relational meanings (e.g., affection), and that these meanings are shared by the majority of humans in a so-called *social meaning model* (e.g., a handshake indicates a formal relationship whereas an embrace suggests an intimate one).

Moreover, Major and Heslin [27] pointed to the importance of the direction of touch: Observers evaluate a person who initiates touch as higher in status, more assertive, more expressive and warmer than a person who receives touch.

In contrast to a social meaning model [26], Constance Classen [38] spoke of sensory (meaning) models which are shared within a culture or society, but are also subject to changes (e.g., a switch from healing touches in medieval times to a strong reliance on technology in modern Western cultures [39]). These models determine the order of the senses within a culture and norms concerning tactile behavior (e.g., who is allowed to touch whom). The relational meaning assigned to touch––with humans or robots––seems therefore limited to a particular cultural environment and time in history (see below for a more detailed discussion of how relational interpretations affect the perception of touch).

Regarding the positive effects of interpersonal touch, work by Tiffany Field revealed that deprivation of touch has negative consequences on individuals’ health, such as sleep disturbances, suppressed immune response, growth restriction, tactile sensitivity, and allergic conditions [40]. Conversely, touching can be useful as a therapeutic tool to foster children’s development, e.g., through massages [41–43].

For adults too, several positive effects of touch on recipients’ well-being have been reported, such as pain and stress reduction [22,44] or prevention of catching a cold [23]. In the latter study, the risk of being infected with a cold was significantly decreased when participants reported being frequently hugged.

One prominent example of the behavioral consequences of touch is the so-called “Midas touch” effect. Crusco and Wetzel [28] coined this term based on the Greek king Midas, who turned everything he touched into gold. The authors observed that waitresses who briefly touched customers on the hand or shoulder as they were returning change received significantly higher tips. The effect was later replicated for tipping [45,46] and for other purchasing behaviors (e.g., shopping time and amount of purchasing [46,47]; amount of impulse purchasing [48]; alcohol consumption [49]).

Moreover, a variety of studies have demonstrated that people are more willing to comply with a request if the person asking a favor uses touch (e.g., participate in a course [32]; help an experimenter after the original experiment [33]; sign a petition [29]; lend a dime [34]; look after a dog [31]).

Furthermore, the initiator of touch and the surroundings have been shown to be evaluated positively. For example, a library clerk and the library itself were evaluated more favorably if the clerk touched students’ hand while handing over their library card [30]. Similar findings have been observed when touch was utilized in other contexts, such as counseling [50], car selling [51] or teaching [52].

In some examples, favorable effects of interpersonal touch occurred after immediate skin-to-skin or flesh-to-flesh contact (e.g., during massages [41–43,53]), while brief touches through clothing (e.g., touch to the shoulder (e.g., [28,45–47]) also evoked positive reactions. Obviously, complex touches such as those used during massages are not easily equatable with mere skin contact. However, since both are related to positive outcomes, we are firstly interested in the impact that brief touches by a robot might have on humans. According to the literature, skin-to-skin contact thus seems not to be a prerequisite for positive effects to arise. Hence, robots that are not covered with synthetic flesh still bear the potential to elicit a positive impact. Also, brief touches are of particular interest since they are realizable with available robotic platforms such as the NAO.

### Explanations for the positive effects of interpersonal touch

According to Fisher, Rytting and Heslin [30], for touch to exert positive effects, it has to convey a message of care and concern and meet the level of intimacy preferred by the recipient. This adds a cognitive-interpretational dimension to the experience of touch. If a positive interpretation and preferred level of intimacy are not given, interpersonal touch is rather undesirable and can result in rejection.

Positive effects of complex interpersonal touching (e.g., massages) have been related to hormonal changes [21,22,44], as well as neurological linkages between skin receptors and pleasant experiences [54]. However, regarding brief touches it is unclear whether positive reactions can be solely attributed to a slight skin contact.

A hormonal response alone, i.e., touch to receptors in the skin trigger a hormonal response that ‘always’ causes favorable effects of touch, cannot explain individual experiences and positive consequences of touch.

Recently, social psychologists [55] published a theoretical mechanistic model to explain how affectionate interpersonal touch may favorably impact the recipient’s well-being. According to their model, receiving affectionate touch can cause (a) an interpretation of touch as a sign of love and care, which leads to relational-cognitive changes (e.g., feelings of security and inclusion; *relational-cognitive pathway*), or (b) immediate neurobiological changes, which do not need reasoning or interpretation (*neurobiological pathway*). These changes––relational or neurobiological––can reduce stress in the short-term, and improve long-term relational, psychological and physical well-being. The model is proposed for receiving touch from a close other, however, research findings concerning touch between strangers [28,30,51] as well as studies on touch with inanimate objects (e.g., a teddy bear [56]) indicate that the explanations might hold for a variety of contacts. It is questionable if touch from a robot can also cause such a positive impact. If a robot is applied in a social context, such as tutoring or counseling, it is highly likely that humans will add an interpretation to touch from the robot in order to make sense of its behavior. Yet, it remains unclear whether the interpretation of the robot’s behavior will be in relational terms or rather technical. Furthermore, it is an open question whether touch from a robot can elicit physiological or hormonal changes, which we do not investigate in the present work. However, related studies indicate that caressing robots has physiological consequence, for instance, lowered blood pressure [3]. Hence, it seems reasonable to assume that touch from a robot can have a positive impact on human well-being, as long as the touch is acceptable in a given situation and interpreted in favorable terms.

Jakubiak and Feeney [55] further highlight the superiority of assigned meanings:

…physical sensations are imbued with meaning, and that meaning can shape the physical experience. (…) the way that individuals think, feel, and behave in their relationships may depend more on their interpretations of the physical and social environment than on the environment itself (p.243).

The subjective experience of touch in a given situation appears thus highly dependent on its interpretation. The interpretation might furthermore depend on the properties of touch, as well as personal, situational, relational and cultural variables [55].

Besides, the effect of touch on compliance––and the same should apply to prosocial behavior and other favorable outcomes of touching––is suspected to be confounded by the stimulation of other senses such as vision and hearing simultaneous to touch [57].

### Technological touch

New sensors and technological developments such as haptic gloves enable the manipulation of virtual objects and tactile contact over distances [58]. In this realm, mediated social touch has frequently been investigated as a means to allow for touch over a distance (see [59] for a review). Also, robots have been applied as a medium to convey touch, e.g., a handshake, between locally distributed individuals (Bevan & Fraser, 2015) [60]. The study revealed that a mediated handshake can increase cooperation during a negotiation task compared to no handshake.

The incorporation of touch into technologies can be regarded as a quantifying process, in which emotions and meanings are treated as being easily mapped onto particular touch gestures. However, this procedure has been criticized for disregarding that touch-affect connections are not easily generalizable [55].

In the following section we focus on immediate touch between humans and robots, where the robot is not a mediator of touch from another human, but an agent on its own.

### Touch in human–robot interactions

In the realm of human–robot interactions, the investigation of humans’ reactions to touch from an artificial but embodied entity is of great interest. Contrary to other physical objects or technological devices, robots, and especially humanoid robots, may be regarded as representing an interface between objects and human beings, since their appearance and behavioral possibilities can be easily interpreted in a social manner. As Walker and Bartneck [61] stated: “Tactile interaction is at the heart of human–robot relationships.” (p. 807), highlighting the capability for tactile interaction (i.e., touch) that distinguishes physically embodied robots from other kinds of artificial entities such as virtual agents.

Previous research in this area, for instance, demonstrated that touching a soft robotic seal covered with fur lowers depression, stress, and pain [2,3,16,62,63]; effects that are also a common result of therapies with living animals. Likewise, Banks, Willoughby and Banks [64] demonstrated that even repeated interaction with a robotic dog that is made of plastic (here: Sony’s Aibo) reduced loneliness in elderly people as effectively as interaction with a living dog. The application of robots in such areas has therefore been termed robot therapy or ‘robotherapy’ [65]. In these cases, the calming effects have been assumed to emerge from touching the robot, e.g., caressing it.

Other researchers considered the impact of mutual or reciprocal forms of touch. For instance, Nie, Park, Marin, and Sundar [66] investigated the effect of handholding and physical warmth in a setting where a human and a humanoid robot (small-size, toylike appearance) watched a horror movie together. During the viewing, the robot either asked the human to hold its hand, which was either cold or warm (with the help of an attached heat pad), or not (no-touch control). Participants reported a higher perception of friendship between them and the robot when they held the warm hand of the robot, but also more fear of the robot compared to the no-touch condition. Moreover, more trust and human-likeness was attributed to the robot after holding a warm robot hand compared to a cold hand or not holding hands at all. Holding hands with a robot thus appeared to change the image participants had of the robot: On the one hand, it increased trust and friendship, but on the other hand, the similarity to a warm human hand seemed to make them feel uneasy. Recently, work by Block and Kuchenbecker [67] supports that individuals favor soft and warm over hard and cold robot hugs.

In a less intimate interaction scenario, Cramer, Kemper, Amin, Wielinga and Evers [68] showed that a toy-like, humanoid robot (Robosapien V2) was evaluated as more reliable and less machine-like by observers if the human–robot interaction included touch compared to no touch. However, the interaction with touch was only favored if the robot’s overall behavior was proactive (i.e., offers help without being asked), whereas a touch-free interaction was favored if the robot’s behavior was reactive (i.e., the robot offered help on demand). Thus, the question of whether touch with a robot is desirable depends on the matching of such behavior to other behaviors of the robot. One shortcoming of this work is that participants were only observers of touch presented in a video recording, which might be qualitatively different from the actual experience of touch [66]. In addition, the meaningfulness of the findings can be criticized, because the video recordings included several forms of touch (i.e., human tapping the robot, robot tapping the human on the shoulder, a hug, a high five), which were not separately investigated. Therefore, the findings do not allow for conclusions on robot-initiated touch. Moreover, interpersonal touch research (e.g., [26,27]) has stressed that each form of touch has its own meaning and effects; thus, each form of touch should be investigated separately before general conclusions can be drawn. Given that almost no studies have focused on robot-initiated touch, we therefore decided to concentrate on analyzing the effects of touch that is initiated by a robot and directed to a human. We believe that it is of particular interest to test whether positive effects of interpersonal touch can be replicated with robots.

With regard to robot-initiated touch, Chen et al. [4,5] demonstrated that participants in the role of a hospital patient in bed experienced instrumental touch (i.e., touch with the purpose of cleaning) as significantly more enjoyable and necessary, and less arousing, than affective touch (i.e., touch with no clear purpose) from a mechanical-looking robot with a towel attached to its end effector. Besides the purpose of touch, the authors varied whether or not touch was preceded by a warning (e.g., “I am going to rub your arm”), and observed higher arousal and a stronger rejection when a warning preceded touch. The findings suggested that a robot-initiated touch with a clear instrumental purpose, but without a prior warning, was the most acceptable in a hospital context. In terms of the behavioral reactions on the part of the participants, the authors did not analyze the video recordings of the experimental sessions in detail, but did include some example pictures showing participants’ facial expressions while the robot was touching them. The reactions ranged from amused (smile, corners of the mouth raised) to skeptical, surprised, or even fearful (raised eyebrows, wide-open eyes). Since arousal (measured by galvanic skin response) increased in all conditions in which the robot touched the participants, it can be concluded that touch evoked positive as well as negative arousal in the participants, corresponding to the facial responses. However, since the reactions appear to be manifold and no information was provided about whether the different reactions varied systematically with the experimental conditions, it remains open which reaction can be expected if a robot initiates touch without any warning. Nevertheless, it can be derived that bodily reactions (facial expressions as well as changes in distance or bodily posture) are interesting variables that should be assessed as a potential consequence of robot-initiated touch. In contrast to the findings by [4], a later experiment that examined functional touch from a humanoid robot (Softbank Robotics’ Pepper) in a similar hospital setup demonstrated that participants reported more safety, comfort and liking of the robot if it announced contact before it touched the participant [69].

Shiomi et al. [6,7] examined the impact of a robot’s touch (Robovie mR2, small-sized, toylike robot with plastic skin) on human motivation. In their experiment, a robot requested that participants perform a monotonous drag-and-drop task under the conditions of active touch (i.e., the robot stroked the participant’s hand), passive touch (i.e., the participant touched the robot’s static hand) or no touch. The results revealed that participants in the active touch condition continued the task for significantly longer. Moreover, compared to those in the no-touch condition, they evaluated the robot as friendlier. In sum, comparable to the interpersonal touch literature (e.g., [30,51,52,70]), active touch from a robot also seemed to elicit favorable evaluations and increased the recipients’ motivation.

The same robot (Robovie mR2) was used in a study examining the positive influence of robot-initiated touch on feelings of unfairness during an ultimatum game [8]. In the touch condition, unfair proposals were offered to participants while a robot touched their arm, while in the no-touch condition, unfair proposals were offered while the same robot did not touch the participants. The results indicated that robot-initiated touch inhibited feelings of unfairness on a neural level (measured via EEG), but no changes in participants’ immediate behavior were observable.

In contrast, Willemse et al. [9] did not report any differences in subjective ratings (e.g., attitude towards the robot, perceived human–robot relationship and perceived human-likeness), in physiological reactions (e.g., heart rate and cortisol), or in participants’ behavior (i.e., compliance with the robot’s request to donate money) after robot-initiated touch from a humanoid robot to participants’ shoulder during the shared reception of a scary movie.

Moreover, the kind and quality of touch a robot initiates were examined more closely. For example, Zheng, Shiomi, Minato and Ishiguro [71] investigated how the perceived intimacy and naturalness of robot-initiated touch depend on the kind of touch a robot applies. Therefore, a female android robot (ERICA) was used that either touched, patted, stroked or grabbed human participants at their hand or forearm [71]. The analyses revealed that patting was rated as significantly more intimate and natural than a simple touch, regardless of the location that was touched. Furthermore, Zamani, Moolchandani Fitter and Culbertson [72] showed that motion parameters such as speed, pauses and force also affect how robot patting touch is experienced by humans (e.g., low speed evokes a higher sense of safety than fast speed). However, the robot in the latter investigation was not an android robot but a collaborative robotic arm equipped with a facial display (Rethink Robotics’ Sawyer). Additionally, Block and Kuchenbecker [67] demonstrated that temperature (cold versus warm) and softness (hard plastic cover versus soft custom-made cover) affect how individuals evaluate hugs with a humanoid robot.

### Summary and objective

In summary, touch research in the realm of human–robot interaction has so far yielded mixed results regarding the question of whether tactile interaction with robots has behavioral and evaluative consequences that are to some extent similar to reactions to animal touch [63,73,74] and also to interpersonal touch [7,8]. However, the majority of studies concentrated on touch directed to robots [3,16,62,73,74] or reciprocal touches [66–68,60], while only a small number of studies have investigated robot-initiated touch [5,7,9,75]. Furthermore, no firm conclusions can be drawn from previous studies on robot-initiated touch, as the studies either relied on the mere observation of touch [68,76], which might be qualitatively different from actual touch, or the researchers implemented robot-initiated touch in a way that required the human to touch the robot first [6,7]. The conditions under which the touch from a robot can elicit positive behavioral and evaluative consequences remain unclear, with some studies pointing to positive effects, e.g., on motivation [7] or feelings of unfairness [8], and others failing to find such effects [9]. Moreover, earlier studies on robot touch did not systematically report on the immediate reaction to touch.

The present work thus focuses on the question whether non-functional robot-initiated touch can elicit positive behavioral and evaluative consequences in actual human–robot encounters, similar to the effects observed in the interpersonal context. To overcome the shortcomings of previous research, we investigated actual touch from a robot in a conversational interaction, which allows for robot-initiated touch without the human having to touch the robot first (cf. [6,7]). In addition, we considered immediate reactions as well as evaluative consequences of touch by means of behavioral observations and self-reported data.

### Hypotheses

Given the still limited body of research on robot-initiated touch, our hypotheses are partly derived from earlier research on interpersonal touch. Initial findings pointing to similar reactions between robot touch and interpersonal touch (e.g., [3,7,8,16]) suggest that this procedure is reasonable.

With regard to behavioral consequences of robot-initiated touch, we consider compliance and prosocial behavior as potential positive consequences.

Research on robot touch has already revealed that active touch from a robot to the hand of a participant increases the participant’s willingness to continue a task [6,7]. However, in the latter study, participants had to touch the robot’s hand in advance, which differs from mere robot-initiated touch. Willemse et al. [9,75] did not observe the hypothesized higher willingness of participants to donate money after they were touched by a robot during the reception of a stressful movie. Given that the interaction context investigated in the present study is not stressful, we assume, similarly to findings from interpersonal touch research, that touch from a robot will increase participants’ willingness to comply with a request and to help another person (e.g., [20,29,31,32]).

**H1:** Individuals who experience robot-initiated touch during a request are more likely to comply with the request than individuals who are not touched.
**H2:** Individuals who experience robot-initiated touch are more willing to subsequently help a person in need than individuals who are not touched.

As no elaborated knowledge on the immediate reactions to robot-initiated touch was reported in previous work, we formulated a research question and observed participants´ behavior in response to touch from the robot:

**RQ1:** What kind of immediate behavioral reactions do individuals who experience robot-initiated touch show?

With respect to relational consequences of touch, in line with Burgoon [26], we hypothesize that an interaction with touch has a different meaning than an interaction without touch. Moreover, since Kleinke, Meeker and La Fong [77] demonstrated that relationships of couples are evaluated as being closer if touch is included, we assume that the presence of touch from a robot will lead to higher perceived closeness. This is in line with the model proposed in [55], which assumes that (affective) touch evokes feelings of security which further causes an increase in closeness and dependence.

**H3:** Individuals who experience robot-initiated touch assign a different relational meaning to the interaction than individuals who are not touched.
**H4:** Individuals who experience robot-initiated touch evaluate their relationship with the robot as closer than individuals who are not touched.

Related to the positive consequences that interpersonal touch has on human well-being, and the positive impact that touching robots has in the realm of robot therapy [16,65], we hypothesize that non-functional touch that is initiated by the robot itself will have positive consequences on the touched individual. In addition, the evaluation of the touching individual (here: the robot [6,7,68]), and the interaction as a whole, should benefit from robot-initiated touch [30,50–52,70].

**H5:** Individuals who experience robot-initiated touch report better feelings (a) during and (b) after the interaction compared to individuals who are not touched.
**H6:** Individuals who experience robot-initiated touch evaluate the robot more favorably than individuals who are not touched.
**H7:** Individuals who experience robot-initiated touch evaluate the whole human–robot interaction (here: counseling) as more positive than individuals who are not touched.

## Materials and methods

The hypotheses were tested in a between-subjects laboratory experiment, in which participants had a conversation with a humanoid robot that either touched them or not. For this purpose, we manipulated the presence of robot-initiated touch (present versus absent).

### Participants

Volunteers were recruited on campus at a European university and received either course credit or monetary compensation. The study was announced as testing a robotic student counselor.

Participants were informed that their interaction with the robot would be video-recorded, and that they could quit the experiment at any time without further justification. To ensure that the experimenter remained blind to the condition, a confederate who operated the robot from a separate room randomly assigned the participants to the experimental conditions. Participants’ sex was balanced across the conditions. In total, *N* = 49 subjects participated in the experiment (*n* = 25 in the touch condition, *n* = 24 in the no-touch control condition). One female participant in the touch condition was identified as strongly touch-avoidant and was thus excluded from the final analyses since she deviated strongly from the means with regard to several dependent measures (lower *need for interpersonal touch* and expertise, higher negative attitudes towards robot before the interaction, more negative subsequent evaluation). This led to a remaining sample of *N* = 48, of whom 26 participants were female and 22 were male, with a mean age of 21 years (range: 18–30 years; *Mean* = 21.15, *SD* = 2.18).

### Ethics statement

The experimental procedure was approved by the university’s local ethics committee belonging to the division of Computer Science and Applied Cognitive Sciences at the Faculty of Engineering of the University of Duisburg-Essen. Written informed consent was provided by all participants. The individual in this manuscript has given written informed consent (as outlined in PLOS consent form) to publish these case details.

### Robot and experimental manipulation

**Fig 1:**
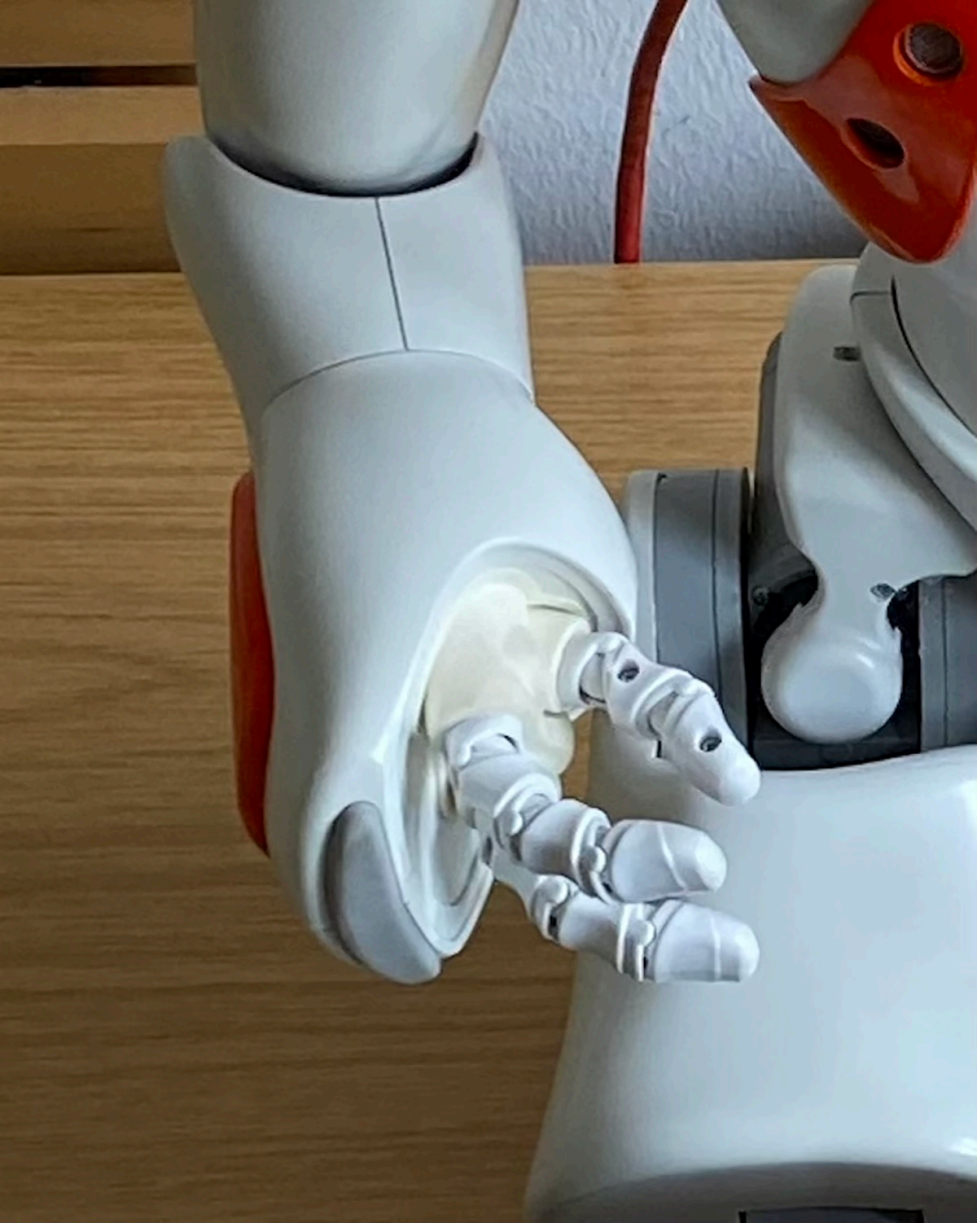
Close-up of the robot’s hand with three fingers.

To study the impact of robot-initiated touch on human subjects, we manipulated the presence of a robot’s touch during a counseling interaction between the robot and students in a laboratory setup. As the robot platform, we chose Softbank Robotics’ humanoid robot NAO. The robot is 22.6 inch tall, weighs 12.1 lb and has 25 DOF. Its body is fully covered with plastic skin. Its hands consist of three fingers resembling two index fingers and a thumb. It is able to execute a thumb-index finger grasp with all three fingers (Fig 1).

**Fig 2:**
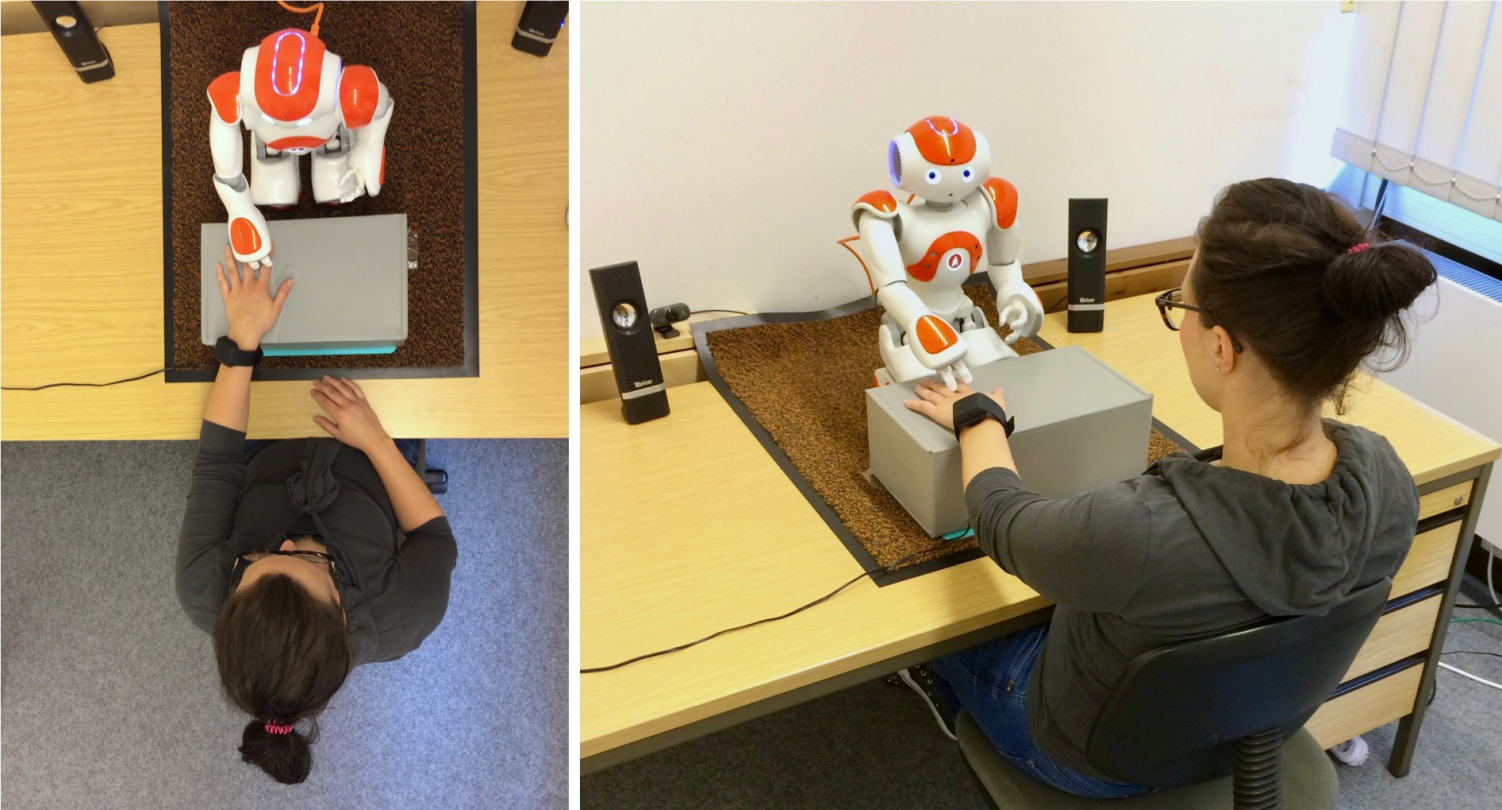
Example picture of the experimental setup showing robot-initiated touch to the back of a participant’s left hand (A) from top view (B) from side view.

Touch was either present (touch condition) or absent (no-touch control condition). The robot’s touch was directed to the back of participants’ left hand (Fig 2), which has been revealed to be a body part accessible to strangers [78]. When the robot initiated touch, the fingers were stretched to result in a position that allowed for the desired touch at participants’ hand (Fig 2). Each time the touch gesture was initiated, the robot leaned forward, reached the participants’ left hand and patted it three times (touch-release, touch-release, touch-release). Afterwards, it retracted its arm and continued the conversation in its initial posture. The arm and finger pose were chosen based on repeated pre-tests with four uninvolved researchers.

To allow for touch without a prior warning or a request to touch the robot in advance, cf. [5,7], we used a cover story to ensure that participants placed their hand in reach of the robot. Participants were told that their skin conductance would be measured to test whether robot counseling is stressful. For this purpose, they had to wear a wristband (Empatica E4) and were asked to place their hand on a box in front of the robot (Fig 2, see also S1 Text). The box was a large, covered shoe box (14 x 8 x 5,5 inch) with an attached cable. The box was filled with books to increase its weight and stability.

The touch was designed to be non-functional and to happen casually. The robot touched the participants four times in the same manner to ensure that the touch was recognized, and to distinguish reactions to initial touch from repeated touch exposure. Comparable to the work of Alagna et al. [50], the robot touched the participants’ hand while posing questions or commenting on the conversation (Table 1). In the no-touch condition, the robot executed the same movements, but with a higher arm angle, so that the robot’s hand did not touch the participants.

**Table 1:** Speech accompanying robot-initiated touch.

Touch sequence	Context	Co-speech
t1	Clarification question	*“Wait*, *please*! *I’m not sure whether I understood you right*. *Could you give an example of what you’ve learned or done exactly during your studies so far*?*”*
t2	Notification of understanding	*“Yes*, *I understand*. *Did you know that most students report that the parties are the best part of studying*? *I can imagine that*, *although I have never been to a party*.*”*
t3	Request for compliance	“*Wait a minute*! *It occurs to me that a course for Business English is taking place in the next semester*. *The course is quite popular and will make a good impression in your CV*. *Are you interested in such a course*?”
t4	Announcement/ end of interaction	*“Okay*, *that’s it for today*. *It was a pleasure talking to you*. *I’m sorry to say that I have many more appointments today*, *let me just ask you a final question*: *If you could choose your dream job*, *what would it be*?*”*

### Procedure

When participants entered the laboratory, they were briefed that they were about to have a conversation with a robot counselor. They were further informed that their conversation with the robot would be video-recorded for post-hoc analyses. Participants were intentionally not informed that the robot might touch them during the conversation. Once they had agreed to take part in the study, the participants were initially asked to complete a pre-interaction questionnaire regarding their personal traits and prior experiences (see Measures). Next, they were instructed to put on the wristband and were guided to the robot. Once they were seated in front of the robot, the experimenter explained how they should place their hand(s) on the box to measure their skin conductance level (note that the box was only a mockup to enable controlled touch from the robot to the participant, and skin conductance was not actually measured). The dialogue and the robot’s movements were scripted and pre-programmed with NAO’s Choreographe Suite to ensure that all participants experienced the same course of the conversation except for the presence of touch. The robot was therefore teleoperated by a confederate in a separate room (a so-called Wizard of Oz setup, [79]) who surveilled the course of the dialogue and decided when the robot should execute a behavior (i.e., movement and co-speech according to the predefined script). After the counseling interaction, the robot asked each participant to move to a separate desk to complete a web-based post-interaction questionnaire. At that moment, the experimenter re-entered the laboratory holding a clipboard with sheets, which the experimenter seemingly dropped accidentally in front of the participant. The experimenter excused herself and picked up one sheet at a time. The participants’ willingness to help pick up the sheets was noted down by the experimenter. Then, the experimenter opened the post-interaction questionnaire on a computer and left the room again. When the participant had finished the questionnaire, the experimenter re-entered the room and followed up with a short interview. Finally, participants were fully debriefed and reimbursed with course credit or monetary compensation.

### Measures

To address the proposed hypotheses and research question, we used objective as well as self-report measures. We used questionnaires in German to collect self-report. English questionnaires were translated to German by a researcher and back-translated by another one to ensure validity. We calculated Cronbach’s α as an estimator of scale reliability. According to [80], values between 0.5 and 0.7 represent moderate, between 0.7 and 0.9 good, and above 0.9 excellent reliability of a (sub-)scale.

#### Immediate reaction to touch

As objective measures, we considered participants’ immediate reaction to touch as collected via video recordings from two different angles (frontal view showing details of the participant’s face, and landscape view showing the interaction between robot and participant from the side). Participants’ reactions visible from the video recordings were inductively coded by one member of the research team (i.e., categories were developed based on the behavior observed in the recordings and the material was iteratively coded accordingly). The coding was repeated by an independent second rater (which was a student assistant), who used the coding scheme developed by the first rater. Interrater-agreement was determined by means of Cohen’s *κ*, which demonstrated excellent agreement (*κ*’s > 0.75) according to [81].

From the frontal camera (webcam), we determined changes in participants’ facial expressions and gaze behavior. From the landscape camera, we noted whether participants moved their hand or body posture when touch occurred. For further analyses, we coded the presence and absence of the identified behaviors of interest (1 = present, 0 = absent), i.e., gaze towards the touched hand (or towards the robot’s moving hand in the control condition; *κ* = 0.83 at t1, 0.93 at t2, 0.87 at t3, 0.87 at t4), smiling (raised corners of mouth with or without open mouth; *κ* = 0.79 at t1, 0.80 at t2, 0.75 at t3, 0.79 at t4)), and eyebrow raise (*κ* = 1 at all times).

#### Compliance

Participants’ compliance with a request from the robot was also determined from the video recordings. For this purpose, during the counseling, the robot asked the participant whether she/he would be willing to take part in a Business English class at university. Participants’ answers were coded as 0 = no, 1 = yes, and ‘missing’ if their answer was ambiguous (e.g., “maybe”).

#### Prosocial behaviour

Participants’ willingness to help after touch from a robot was tested immediately after the interaction, when the experimenter—seemingly accidentally—dropped a pile of papers she was carrying in front of the participant. This procedure has previously been used to study the impact of interpersonal touch on helping [20]. The experimenter then slowly lifted one sheet after another to give the participant a chance to help. We noted whether the participant helped by picking up at least one sheet (1 = yes) or not (0 = no).

### Self-report measures

All self-report measures were collected via questionnaires, which were incorporated into a web-based survey (https://soscisurvey.de) with the benefit of reducing transcription errors. Items were rated on a 5-point Likert-type scale ranging from 1 = “strongly disagree” to 5 = “strongly agree,” unless otherwise stated.

The survey was divided into two sections: one to be completed before the interaction with the robot and one to be completed afterwards. The first part asked about participants’ attitudes and anxieties towards robots, prior experiences and demographic information. The second part—which was completed after the interaction—asked about participants’ feelings as well as their evaluation of the robot and the interaction as a whole. In addition, we included several manipulation check questions and asked about participants’ general attitude towards touch.

#### Attitudes towards robots

Participants’ attitudes towards robots in general were assessed by means of the 14-item version of the *Negative Attitudes toward Robots Scale* (NARS: [82]). The scale consists of three subscales that measure negative attitudes towards: 1) situations of interactions with robots (NARS S1, six items, *Cronbach’s α* = .71, *M* = 2.64, *SD* = 0.70), e.g., “I would feel very nervous just standing in front of a robot,” 2) social influence of robots (NARS S2, five items, *Cronbach’s α* = .60, *M* = 3.04, *SD* = 0.67), e.g., “I feel that in the future, society will be dominated by robots,” and 3) emotions in interaction with robots (NARS S3, three items, *Cronbach’s α* = .66, *M* = 3.35, *SD* = 0.74), e.g., “I feel comforted being with robots that have emotions (reverse-coded).”

#### Robot anxiety

Interaction-specific expectations and fears with regard to robots were assessed using the *Robot Anxiety Scale* (RAS: [83]). The scale consists of eleven statements that refer to three dimensions of robot anxiety: *anxiety towards communication capabilities of robots*, e.g., “I am concerned whether the robot might not understand difficult conversation topics” (RAS S1: two items, *Cronbach’s α* = .65, *M* = 3.41, *SD* = 0.93), *anxiety towards behavioral characteristics of robots*, e.g. “I am concerned what kinds of movements the robot will make” (RAS S2: four items, *Cronbach’s α* = .92, *M* = 2.48, *SD* = 1.11), and *anxiety towards discourse with robots*, e.g., “I am concerned how I should respond when the robot talks to me” (RAS S3: four items, *Cronbach’s α* = .80, *M* = 3.20, *SD* = 0.83).

#### Prior experience and expertise

Participants’ prior experience with robots was assessed on a 5-point scale from 1 = “never” to 5 = “always”. Their average experience with robots at work (*M* = 1.17; *SD* = 0.38) as well as in their leisure time (*M* = 1.19; *SD* = 0.39) was low.

Expertise was measured with six items, e.g., “I am robot literate”, “I am interested in new technologies”, “I can easily deal with new technologies (e.g., smartphones, tablet computers, etc.)” The items were rated on a 5-point Likert-type scale ranging from 1 = “fully disagree” to 5 = “fully agree” and were collapsed into one single score: *expertise in dealing with new technologies*, which showed good reliability (*Cronbach’s α* = .80; *M* = 3.07; *SD* = 0.59).

#### Need for interpersonal touch

Participants’ general attitudes towards interpersonal touch were gathered via the *Need for Interpersonal Touch Scale* (NFIPT: [84], 20 items, *Cronbach’s α* = .86; *M* = 3.51; *SD* = 0.54). To avoid a priming effect, the questions were presented retrospectively (after the interaction).

#### Measuring the relational meaning of touch

We used the *Relational Communication Scale* (RCS: [26,85]) to assess the meaning participants assigned to the robot’s touch. The statements from the RCS were reformulated to match the participants’ first-person perspective in a human–robot interaction situation, e.g., “The robot tried to establish common ground with me”, “The robot acted like it was more powerful than me.” Based on the literature, the 12 items were summarized into four subscales: *affection* (six items, *Cronbach’s α* = .80), *detachment*, *composure* and *dominance* (two items each, *Cronbach’s α’s* < .45). Scales with unacceptable alphas (below 0.5, see [80]) were not considered further. Mean ratings for *affection* demonstrated overall that participants experienced the robot’s general behavior as showing affection towards the participant (*M* = 3.23, *SD* = 0.83).

#### Perceived closeness

To determine how close participants perceived their relationship with the robot to be, we used the *Inclusion of the Other in the Self Questionnaire* (IOS: [86]). The IOS is a pictorial scale that depicts relationships, originally between two persons (person A and B), by means of seven pairs of overlapping circles. We adapted the scale to our scenario and replaced ‘person B’ with ‘robot’). The circles were introduced by the question “How close would you describe your relationship with the robot to be?” The pictures depict increasingly close relationships, starting with separated circles, which we coded as 1 = “not close” and moving to more or less completely overlapping circles coded as 7 = “very close.” The scale has already been applied in other HRI studies (e.g., [75]). Regardless of the condition, the relationship with the robot was judged as rather distant (*M* = 1.96, *SD* = 0.87).

#### Emotional state

Participants’ emotional state during the interaction was assessed using the *Self-Assessment Manikin* (SAM) [87], which consists of three pictorial scales depicting emotional states based on the dimensions *valence*, *arousal* and *dominance*. Participants were asked to indicate how good, aroused and dominant they felt during the interaction with the robot. In addition, we asked about participants´ affective state after the interaction (“How do you feel right know?”) using the *Positive and Negative Affect Schedule* (PANAS: [88]). The instrument consists of 20 adjectives describing *positive affect* (10 items, e.g., active, excited; *Cronbach’s α* = .83; *M* = 3.06; *SD* = 0.63) and *negative affect* (10 items, e.g., hostile, nervous; *Cronbach’s α* = .86; *M* = 1.44; *SD* = 0.50).

#### Evaluation of the robot

The evaluation of the robot was based on characteristics that might have been affected by touch, i.e., “warm”, “human-like” (see [68]), “sympathetic” and “eerie” (see [66]). The items (with the exception of “eerie”), were collapsed into one reliable scale *robot evaluation* (three items, *Cronbach’s α* = .73, *M* = 2.92, *SD* = 0.97). Eeriness was rated low overall but with high deviations from the mean (*M* = 1.75, *SD* = 1.06). Additionally, we measured the robot’s attractiveness by means of the *Interpersonal Attraction Scale* [89], with the subscales *social attraction* (five items, e.g., “The robot could be a friend of mine”, *Cronbach’s α* = .68; *M* = 2.55, *SD* = 0.85), *physical attraction* (four items, e.g., “The robot is quite handsome”, *Cronbach’s α* = .63; *M* = 2.39, *SD* = 0.83) and *task attraction* (five items, e.g., “The robot is a good problem solver”, *Cronbach’s α* = .76, *M* = 3.35, *SD* = 0.71), adapted to evaluate the robot. Alpha values below .7 for social and physical attraction indicate only moderate reliability.

#### Evaluation of the interaction

In line with Burgoon and Walther [90], we assessed the general evaluation of the conversation using four items tailored to the human–robot interaction situation, e.g., “Most people would like to interact with this robot”, “I enjoyed the conversation with the robot.” Negatively worded items were reverse-coded before all items were collapsed into a reliable *general evaluation* scale (*Cronbach’s α* = .80, *M* = 3.56, *SD* = 0.82). Moreover, we asked participants about their satisfaction with the robot counseling by means of the *Interpersonal Communication Satisfaction Inventory* [91]. The inventory consists of 19 statements regarding interpersonal communication, e.g., “I was very dissatisfied with the conversation” or “The other person expressed a lot of interest in what I had to say.” We adapted 16 of the statements to our context and replaced “other person” with “the robot” if necessary. Again, negatively worded items were reverse-coded, and all items were collapsed into one score (*Cronbach’s α* = .86, *M* = 3.15, *SD* = 0.63).

#### Manipulation check

As a manipulation check, we asked all participants whether the robot touched them during the conversation, how often and on which part of their body. The manipulation check was successful, as only participants in the touch condition indicated that they were touched by the robot, and they further confirmed that they experienced touch to their left hand several times during the interaction.

#### Evaluation of touch

To achieve a deeper understanding of how participants perceived and evaluated the touch from the robot, a five-point semantic differential was administered to participants in the touch condition only. The differential consisted of eight pairs of adjectives that describe the touch experience (e.g., unpleasant/pleasant, expected/unexpected).

#### Further collected data not relevant for the present paper

We additionally collected data on individuals’ need to belong [92] and loneliness [93], which were not analyzed for the purpose of the present paper.

## Results

The results section is divided into qualitative findings based on video-recordings and behavioral observations from the human–robot interactions, and quantitative findings based on questionnaires. An alpha level of .05 was applied for all inferential statistics. As effect sizes we calculated coefficient *φ* for frequency comparisons (i.e., χ^2^ tests) and Cohen’s *d* for mean comparisons (t-tests). According to Cohen (cited after [94] p. 41), a *φ* below 0.3 indicates a small, between 0.3 and 0.5 a medium, and above 0.5 a large effect. Regarding Cohen’s *d*, values below 0.5 indicates a small, between 0.5 and 0.8 a medium, and above 0.8 a large effect.

### Behavioral analyses

As objective measures, we considered a) participants’ immediate reaction to touch and b) their compliance with a request from the robot. In addition, c) we observed participants`helping behavior after the interaction in order to determine the influence of robot-initiated touch on these variables.

#### Immediate reaction to touch

To answer RQ1, content analyses of the crucial segments in which the robot touched the participant’s hand in the video recordings (t1 –t4, and the respective moments in the no-touch condition) were conducted. The analyses revealed that overall, participants’ reactions to robot-initiated touch were not dismissive. None of the participants pulled their hand away or told the robot to stop when the touch happened. The majority of the participants directed their gaze to the touched hand (*n* = 22 at t1, *n* = 21 at t2, *n* = 19 at t3, *n* = 19 at t4 out of 24 participants in the touch condition). The gaze behavior indicated awareness of the touch and that increased attention was paid to the location of touch. However, similar reactions to the robot’s hand and arm movement were observed in the control condition, showing that the increased movement alone was sufficient to direct participants’ attention.

Negative reactions to the robot’s touch were scarce. In two cases, the participants raised their eyebrows when the robot touched their hand at the first touch instances (t1 and t2, case 10 and 16, both female). However, one participant in the control condition likewise raised his eyebrows while monitoring the robot’s movement in the touch-equivalent situation (t1, case 38, male).

**Fig 3:**
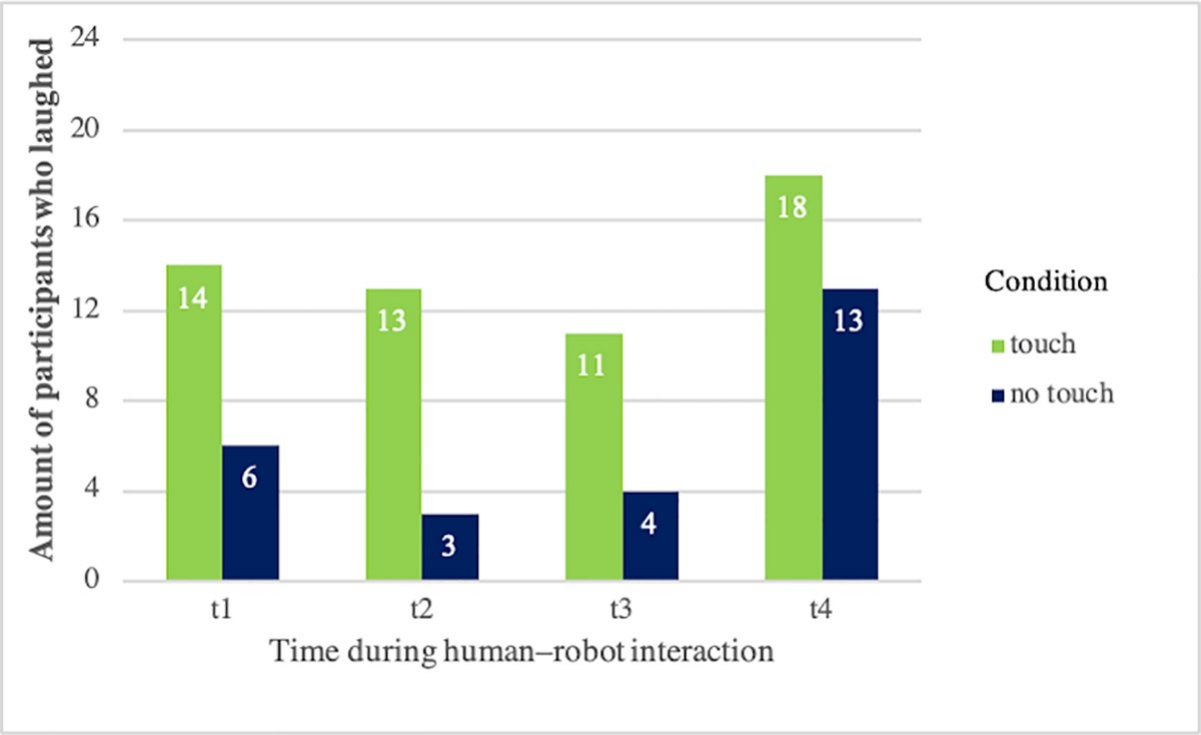
Bar charts for the number of laughing participants at times t1–t4 per condition.

The other participants reacted positively to the touch, with smiling and laughing. In 55 out of 96 possible instances (i.e., 24 participants x 4 times), participants smiled or laughed during or shortly after touch from the robot. In contrast, participants in the control (no-touch) condition only laughed in 26 instances. To test whether smiling and laughing was related to robot-initiated touch, or whether it happened accidentally, we compared the presence of smiling and laughing at each touch instance t1 –t4 between the touch and no-touch condition (Fig 3).

Two-tailed Pearson Chi^2^ test revealed significant differences with medium effect sizes in the presence and absence of laughing between the conditions: More laughing was observable in the touch condition at t1 [(*χ*^*2*^ (1, *N =* 48) = 5.49, *p* = .002, *φ* = .34], t2 [*χ*^*2*^ (1, *N =* 48) = 9.38, *p* = .002, *φ* = .44] and t3 [*χ*^*2*^ (1, *N =* 48) = 4.75, *p* = .029, *φ* = .31], whereas the difference was not significant at t4 (*p* > .05).

#### Compliance

To test whether participants were more willing to comply with the robot’s request to join a course on Business English after touch (H1), we compared the frequencies with which they answered “yes” or “no” between the conditions by means of Pearson’s Chi^2^ square test. Participants who gave ambiguous answers (e.g., “maybe,” “I don’t know”, *n* = 6) were excluded from this analysis.

In support of H1, the test demonstrated that participants who were touched by the robot while it was announcing the course were significantly [Pearson *χ*^*2*^ (1, *N* = 42) = 5.08, *p* = .02, *φ* = .35] more compliant (63%, *n* = 17 out of 21 answered ‘yes’) than participants in the no-touch condition (37%, *n* = 10 out of 21 answered ‘yes’).

#### Helping

Concerning participants’ willingness to help the experimenter after touch from the robot (H2), we compared the number of participants who helped the experimenter to pick up dropped sheets of paper (helped / did not help) between the conditions. Due to errors during the dropping of sheets (e.g., sheets dropped in an unintended direction, out of participants’ reach), data from two of the participants were excluded from this analysis. The frequencies for helping revealed that more participants in the touch condition were willing to help the experimenter (59%, *n* = 13 out of 23) compared to participants in the no-touch condition (41%, *n* = 9 out of 23). However, the difference was not significant according to Pearson’s χ^2^ test (*p* > .05) [*χ*^*2*^ (1, *N* = 46) = 1.39, *p* = .24]. H2 was therefore not supported.

### Evaluation of touch

**Fig 4:**
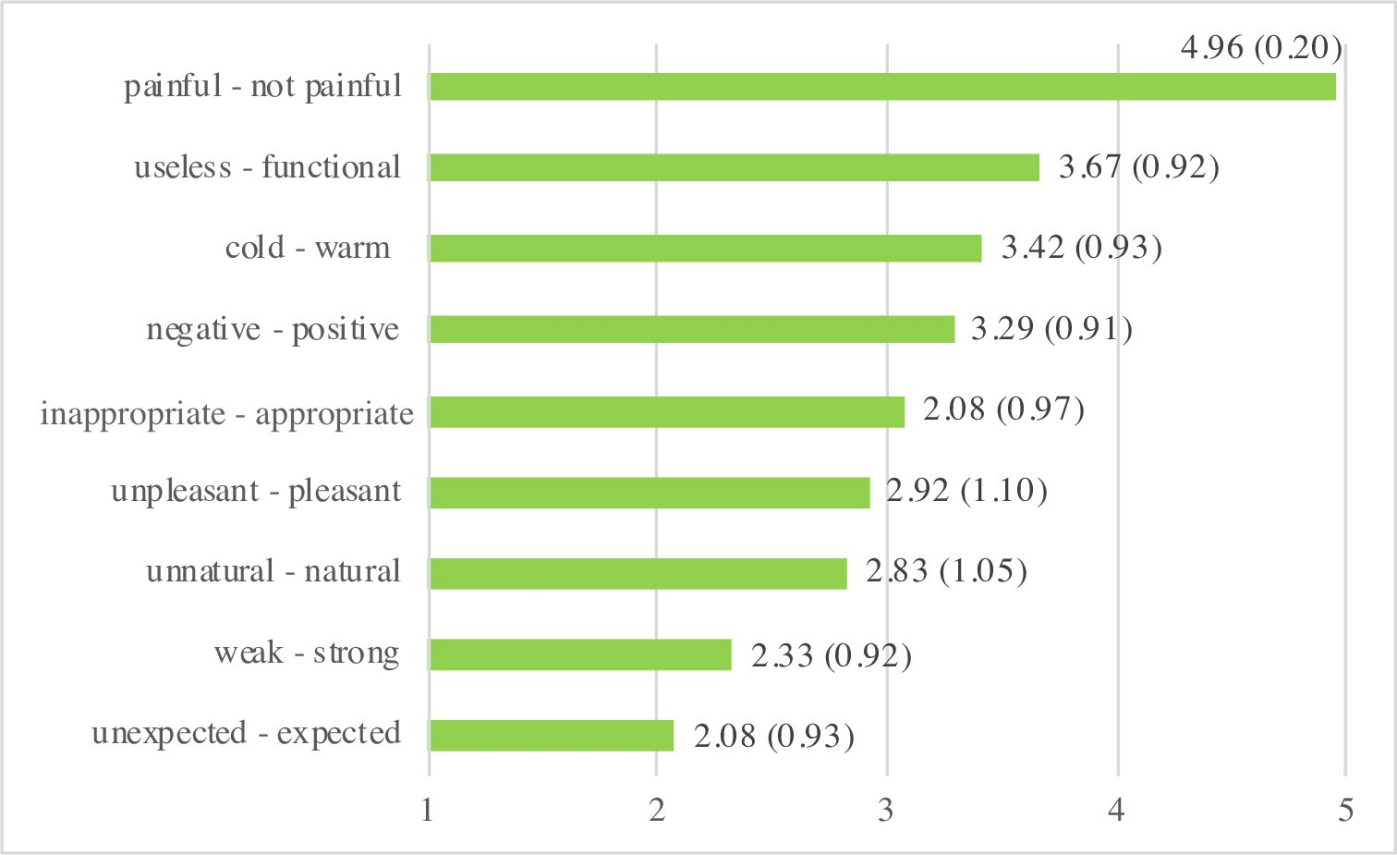
Mean ratings and standard deviations (in parentheses) for the evaluation of touch from the robot for participants in the touch condition (n = 24).

To better understand participants’ subjective experience of robot-initiated touch in the touch condition, we asked them to reflect on their perception of touch based on different adjectives. The mean ratings as summarized in Fig 4 reveal that the touches from the robot were perceived as functional, warm, positive, appropriate, pleasant, natural, weak and not painful. Additionally, the average ratings show that touch from the robot was rather unexpected (Fig 4).

### Subjective ratings depending on the presence of touch

Regarding the subjective measures, we compared means using t-tests after having checked the influence of person characteristics, i.e., need for interpersonal touch, expertise, negative attitudes towards robots (NARS S1, S3) and robot anxiety (RAS S1 and S3), which were correlated with (several) dependent measures. Since no significant differences in the main effects emerged when comparing analyses with covariances with those without, we only report the results of independent sample t-tests instead of ANCOVAs.

#### Observed relational meanings of robot’s touch

In terms of the relational meaning participants attributed to the robot’s behavior, we compared the mean ratings for the subscale affection of the Relational Communication Scale. In line with H3, higher affection was reported after touch, but the difference was not significant (Table 2). Thus, H3 was not supported.

**Table 2:** Descriptive statistics and results of t-test for all subjective measures.

	Condition							
	no touch		touch		One-tailed t-test			
Dependent measure	*M*	*SD*	*M*	*SD*	*t*	*df*	*p*	*d*_*Cohen*_
Perceived affection	3.13	0.79	3.33	0.86	-0.87	46	.194	0.24
Closeness	2.00	0.89	1.92	0.88	0.33	46	.373	-0.09
Valence	3.67	0.82	4.08	0.78	-1.81	46	.038	0.51
Arousal	2.21	0.83	2.13	0.90	0.33	46	.371	-0.09
Dominance	3.33	1.09	3.63	0.92	-1.00	46	.162	0.30
Positive affect	3.02	0.58	3.10	0.69	-0.41	46	.343	0.13
Negative affect	1.57	0.59	1.30	0.35	1.92	37.21	.032	0.56
Robot evaluation	2.94	1.00	2.90	0.97	0.156	46	.442	-0.04
Physical attraction	2.28	0.87	2.50	0.80	-0.90	46	.185	0.26
Social attraction	2.64	0.92	2.46	0.79	0.74	46	.232	-0.21
Task attraction	3.35	0.72	3.36	0.72	-0.04	46	.484	0.01
General evaluation	3.52	0.86	3.59	0.80	-0.30	46	.382	0.08
Communication satisfaction	3.17	0.77	3.12	0.47	0.31	46	.379	-0.08

To achieve a deeper understanding of participants’ interpretations of the robot-initiated touch, besides their ratings on the RCS, we analyzed the statements which participants in the touch condition made during the post-interaction interviews.

The statements show that the touch was actually interpreted as a sign of affection by some participants:

“I liked it when the robot touched my hand. It tried to establish a bond I guess.” (female, 22)
“In the moment it appeared quite natural, … as a person that wants to calm and encourage me.” (male, 21)
“… I understood why it should be like that − to calm me − but it was peculiar (laughs) because he [the robot] only has three fingers.” (female, 20)

Some participants interpreted the touch as functional in terms of supporting the robot’s statements:

“I noticed that it [touch] always happened to support speech.” (male, 23)
“He reached out with his hand to emphasize his statements, …” (male, 20)

By contrast, other participants were uncertain about the meaning of touch from the robot:

“Unexpected. When it happened the second time, it was ok, but the first time I was wondering why he’s doing that” (female, 23)

One participant even reported that she was uncertain whether the touch was intended or a technical failure:

“First I was terrified. I thought ‘Oh it is broken’. But then I noticed that it was intended.*”* (female, 21)

#### Perceived closeness

The results for the IOS revealed slightly higher ratings for closeness in the no-touch condition (Table 2), contrary to H4. However, the difference was small and not significant, thus yielding no support for H4.

#### Emotional state during and after touch

Regarding participants’ feelings during the interaction, they reported higher positive emotions (*valence*) and more dominance in the touch condition compared to the no-touch condition (Table 2). Instead, arousal was rated lower in the touch condition. A comparison of the means revealed that the difference was only significant for valence: Participants who experienced touch from the robot during counseling reported feeling significantly better during the interaction than those who had a touch-free conversation. In line with this, more positive, and less negative, states were reported after the interaction that included touch from the robot, although only the difference for negative affect was significant (Table 2). According to Cohen (cited after [94] p. 41), the observed differences for valence and negative affect resemble a medium effect. The results lend partial support for H5.

#### Evaluation of the robot

The evaluation of the robot in terms of warmth and sympathy was unaffected by touch. On average, the ratings were high (>2.8 on a 5-point scale, Table 2). The robot’s attractiveness in terms of physical, social and task attraction also did not differ significantly according to the presence or absence of touch. Moderate to high mean ratings indicate that overall, the robot was evaluated as attractive, whereas the highest ratings were observable for task attraction (Table 2). The findings contradict H6, which assumed a more favorable evaluation of the robot in the touch condition.

#### General evaluation of the counseling interaction

Regarding the impact of robot-initiated touch on the situation as a whole, i.e., the general acceptance of the interaction (*general evaluation*) and the satisfaction with the conversation (*communication satisfaction*), the evaluations were on average favorable (means above 3 on a 5-point scale, Table 2). However, we did not find any significant differences caused by touch, as the small differences between the means already indicate. In summary, the results do not support better evaluations of the interaction after touch from the robot, as was hypothesized in H7.

## Discussion

In the present study, we aimed to test whether non-functional robot-initiated touch during a conversation can result in similar behavioral and evaluative consequences to interpersonal touch. For this purpose, we conducted an experimental study in which participants were actually touched on the hand by a humanoid robot during a student counseling conversation. The study extends previous research due to 1) the rigorous investigation of (seemingly spontaneous) robot-initiated touch to participants’ hand without prior announcement, 2) the testing of hypotheses theoretically derived from a large body of research on interpersonal touch, and the qualitative analysis of 3) the immediate behavioral reactions, and 4) the meaning individuals assign to robot-initiated touch based on video recordings and interviews.

In the following, we revisit the results and interpret them in light of the hypotheses as well as related research.

### Immediate reactions to robot-initiated touch

The question of how individuals actually react to robot-initiated touch (RQ1) was addressed by analyzing the video recordings from the interactions, which showed that none of the participants pulled their hand or body away when the robot touched them. Most participants remained in their position, while changes in their facial expressions were observable. Overall, participants appeared to be quite content when receiving touch from the robot (here: patting the back of their hand). However, it might be suspected that this finding is attributable to the experimental instructions to keep the left hand on the box to ensure accurate recording of the physiological data, which may have restricted participants’ reactions to robot-initiated touch. Nevertheless, this also brought about higher experimental control: If participants had been free to place their hand wherever they wished and had thus missed the touch from the robot, the comparability between participants would have been reduced, which would in turn have altered the overall experience.

Moreover, the majority of the participants followed the robot’s hand movements with their gaze when they were touched, or even when the robot merely gesticulated in the air (no-touch condition). When touched, most participants reacted by smiling and laughing. It is noteworthy that several participants seemed to show a delayed reaction: When the robot started touching, their attention was first directed at the hand, and then back at the robot’s face, and the laughing often started a few seconds later. In some cases, it seems as if participants first wondered what was going on before reacting positively. Following the initial surprise, favorable reactions emerged, as reflected in highly positive evaluations of the touch as warm, functional, positive, and not painful. The positive reactions might also be a result of the overall friendly appearance of the small humanoid robot covered with plastic. Other reactions can be expected if a taller, or more mechanical-looking robot would touch participants. Other types of robots might further execute more complex touches than just tapping human participant’s hands. Future studies should thus consider replicating our findings with other robot types.

The comparison of the conditions further demonstrated that touched participants laughed significantly more during the first, second, and third touch sequence, but no difference emerged at the fourth touch sequence. A closer look at the content of the conversation revealed that during the final touch instance, the participants seemed to be especially amused by the robot’s co-speech, which was as follows (see Table 1, co-speech at t4):

Oh, I’m sad to say that our time is running out *[looks at its wrist*]. I could have told you so much more. The experimenter will hand you all necessary information that could be important to you. *[pause–starts the initiation of touch or gesticulation in the air]* Okay, that’s it for today (…)

These typical human-like behaviors, e.g., pretending to look at a watch and to have a busy day, might have amused the participants independently of the presence of touch. As a result, the conversational content during the final touch sequence might have overridden the effect of touch.

Furthermore, this might have caused equal evaluations of the robot and the whole interaction in the questionnaires, because the positive experience at the end of the interaction is most likely to be remembered retrospectively. To avoid such an influence, follow-up studies should balance the co-speech content and consider allowing participants to narrate their experience continuously during the interaction in order to obtain immediate evaluations of each touch instance instead of asking for an overall assessment retrospectively.

This observation also supports the notion that touch is not a unimodal sense that can be regarded in isolation from other senses [95]. Hence, understanding how affective touch is experienced with robots is a complex endeavor that needs to take the interplay of certain characteristics of the interaction, such as co-speech, into account [96].

### Consequences of robot-initiated touch

Once we had established that the participants were amenable to robot-initiated touch to their hand and evaluated the touch experience as rather pleasant, we reconsidered how the experience of touch affected other behaviors (namely compliance and helping) and the evaluation of the robot and the conversation.

#### Compliance

As compliance is one behavioral reaction to touch that has been frequently investigated in the realm of interpersonal touch research, we therefore programmed the robot to request participants’ interest in a course while touching their hand. Our analysis of participants’ reactions to the robot’s request demonstrated that participants who were touched were indeed more likely to comply, i.e., showed more interest in the course, than participants who were not touched, as hypothesized in H1. This finding resembles that of Shiomi et al. [7], who reported stronger efforts to fulfill a task after active touch from a robot. In their work, participants first had to touch one hand of the robot before the robot was able to stroke the participant’s hand. Our findings reveal that a positive impact of touch on compliance can also be elicited without asking participants to touch the robot beforehand.

Furthermore, our observation is in line with findings from the interpersonal touch literature that report stronger compliance after touch (e.g., [29,31,33,51]). An explanation for this phenomenon is that when making her or his request, the touching person is perceived as being in genuine need and as trusting the touch recipient [54]. The same could apply to the robot in the present study: Participants might have unconsciously perceived the touch from the robot as a sign of trust, which in turn increased compliance. As in the interpersonal context [57], we cannot rule out that confounding variables such as the robot’s co-speech and its friendly, shining eyes affected the experience of touch, because the sensation of touch does not happen in isolation.

Nevertheless, robot-initiated touch seems to reinforce a request, which then increases compliance. A beneficial strategy may therefore be to purposely use robot-initiated touch to encourage individuals, for example, to exercise or to take their medication. Nevertheless, it can be questioned whether the robot’s request in the present study was sufficiently strong to measure compliance. Future studies could consider using stronger indicators of compliance, such as asking participants to sign a form to register for a course instead of only asking whether they are interested in the course. It is remarkable, however, that even though compliance was measured with respect to a low-level request, participants who experienced touch behaved differently than those who did not experience touch. It is furthermore remarkable that simply tapping the back of participants’ hands showed such an effect. Involving more complex and prolonged touching from a robot might increase engagement and compliance anymore.

#### Prosocial behaviour

Contrary to the hypothesized greater willingness to help after touch (H2), we found no difference between the touch and no-touch conditions. Participants in the touch condition were almost equally as likely to help as they were not to help, and the same applied for participants in the no-touch condition. The willingness to help thus appears to be dependent on factors other than the presence of robot-initiated touch. This is in line with earlier observations, who reported no differences due to robot-initiated touch on prosocial behavior (i.e., willingness to donate money to the Red Cross [9], or money donated to robotics research and extra time spent in the laboratory [75]). However, the findings are in contrast to earlier observations from interpersonal encounters, which revealed more helping after touch (e.g., [20,33]).

A substantial difference between the present study and studies on the effects of interpersonal touch on helping is that in the latter studies, the participants were touched by the person in need. In the present study, by contrast, the participants were touched by the robot, whereas the experimenter needed help later on. Consequently, it is possible that the participant would have helped the robot more often after touch (which was not tested), but that the effect did not transfer to the experimenter.

#### Relational interpretation of touch

As interpersonal touch research demonstrated that touch encompasses a relational meaning [26], and Jakubiak and Feeney [55] highlight that the interpretation further shapes the experience of touch, we also asked participants about their relational interpretations of the interaction with the robot, which has not been considered in previous studies. Contradicting H3, we did not observe differences in perceived affection depending on the presence of touch.

It has to be noted, however, that the interpretations of robot-initiated touch as measured with the Relational Communication Scale did not result in reliable subscales. Thus, relational interpretations of detachment, composure and dominance were not further regarded in the analyses. On the one hand, this could imply that these dimensions are less important in human–robot interactions, but on the other hand, it is possible that the items were not adequate to assess relational interpretations of a robot’s behaviors.

Overall high ratings for perceived affection (above 3 on a 5-point scale) might be a result of the robot’s conversational behavior, which was polite and understanding in both conditions. Since the items on perceived affection only ask about general perceptions of the interaction partner, e.g., “*The robot was interested in what I had to say*,” it is possible that the conversational behavior alone resulted in the perception that the robot showed affection and interest in the participants, and that touch only added a minimal increase. This is reflected in slightly higher mean ratings after touch; however, the difference was not statistically significant.

Besides the ratings on the Relational Communication Scale, we considered participants’ interpretations of touch as they described it in the post-interaction interviews. The statements indicate that many participants interpreted robot-initiated touch as a calming gesture that was included to establish a bond between the robot and the participant. However, others interpreted it rather functionally in terms of an action that underlines the robot’s speech. Some participants were even unsure about the meaning of touch, why it was present, whether it was intended or even a technical failure. The findings suggest that the individual interpretations and assigned meanings of touch should be considered in future research as potentially influencing the impact of robot touch. This is especially important if the assigned meaning shapes the physical experience of touch as [55] assume. Furthermore, alternative approaches to assess the meaning that is assigned to robot-initiated touch should be considered, since the Relational Communication Scale alone does not appear to be adequate to fully understand humans’ interpretations of robot touch.

#### Perceived closeness

With regard to the perceived relational closeness, we did not find significant differences between touch and no-touch condition, as assumed in H4. This finding suggests that earlier observations from interpersonal touch studies that touching individuals are perceived as having a closer relationship [77] do not transfer to human–robot interaction. However, these results stem from field observations of touching individuals, whereas the present work examined the direct effect of actual touch between the interaction partners. As such, the contradictory findings might not only result from the difference between interpersonal touch and human–robot touch, but might also be affected by the perspective of the evaluator of closeness (i.e., observer versus active participant [18]). In the realm of mere observations of human–robot touch, Cramer and colleagues [68] reported no difference in the perceived closeness which observers assigned to the relationship of a human and a robot that either did or did not touch each other. However, as remarked above, it is unclear which form of touch determined the evaluation, as different forms were presented in the video recordings.

Admittedly, it needs to be noted that overall, closeness ratings in the present investigation were quite low (means of 1.92 and 2.00 on a 7-point scale). It might be suggested that the pictorial representation of the human–robot relationship by means of circles was ambiguous to the participants. Although the scale has been shown to be highly reliable in interpersonal contexts [86], it is unclear whether the instrument also applies to human–robot interactions. So far, one study demonstrated that the scale was useful to measure closeness with a robot after a phase of bonding [75]. Here, the IOS was administered before and after the manipulation took place. This could also be considered in future work to compare ratings before and after touch. Alternatively, it is possible that the participants felt close to the robot, but denied this when asked, because it might have seemed inappropriate to confess a close relationship with a robot, especially in a laboratory during an experiment [11]. In summary, more research is needed to draw conclusions on the impact of robot-initiated touch on closeness. Especially, the applicability of the IOS scale needs further validation, and alternative measures such as the perceived friendship (see: [75]) should be considered to assess perceived closeness in future work.

#### Emotional reactions to robot-initiated touch

Since work on robot therapy [16,62,97,98] demonstrated beneficial effects of touch on individual well-being, it was hypothesized that robot-initiated touch has a positive impact on participants’ emotional state during and after the interaction (H5). In line with this assumption, robot touch yielded significantly higher positive affect during the interaction, and less negative affect after the interaction. Consequently, participants who were touched by the robot indicated that they felt better during the conversation and reported lower negative affect subsequently. This implies that robot-initiated touch indeed improved participants’ emotional state in comparison to the same interaction without touch. Since the results on relational meanings of touch did not show higher perceived affection, the question arises of what caused this difference, if participants in the touch condition did not perceive more affection. On the one hand, touch might have caused interpretations (or relational-cognitive changes, see [55]) that remained unconscious, or which were different from what was measured on the dimension of affection (example item: “The robot showed affection towards me”). According to participants’ statements after the interaction, the robot’s behavior did not remain uninterpreted. In fact, several statements support the notion that touch was regarded as a calming or bonding gesture. But, in contrast, some participants reported uncertainty about the meaning of touch and were unsure whether it was a technical error. According to the theoretical assumptions, those should not have experienced touch as favorable as others.

On the other hand, the simple touch from the robot (in combination with its speech) could have had a calming effect [22] (the neurobiological pathway in [55]), which in turn increased participants’ positive feelings. However, as we did not measure physiological arousal or hormonal changes in the present study, future research is necessary to investigate whether robot-initiated touch indeed impacts these variables. Moreover, the touch from the robot might at the same time have activated neural linkages that associate interpersonal touch with a pleasant sensation [54]. To test this explanation, future investigations should consider employing brain imaging methods and compare neural activities when participants are touched by a robot in comparison to touch from a human.

#### Evaluation of the robot and the interaction

Contrary to the prediction that the robot and the conversation would be more favorably evaluated in the touch condition, no such differences were observed. Rather, the evaluation of the robot (H6) and the satisfaction with the interaction (H7) were equally favorable in both conditions. Again, these results create the impression that something other than touch alone was responsible for the exceedingly positive evaluation of the robot and the conversation. Statements from the post-interaction interviews gave some hints that all participants enjoyed the interaction because they were impressed by the robot’s ability to understand natural language, and to elaborate on their answers. It is thus conceivable that the high ratings reflect a ceiling effect caused by the overall positive impressions of the robot, which might have prevented differences caused by touch from becoming distinctly visible in the ratings. Overall, the behavioral differences regarding laughing and compliance appear even more surprising given that the reported subjective experiences did not differ a great deal—especially as behavioral reactions cannot be explained by differences in evaluation.

## Limitations

Since the present experiment was conducted with undergraduate students at a European university, who had moderate to high technological expertise, the generalizability of the findings to other cultures and populations is restricted. Although cultural differences were out of scope of the present research, it is vital to notice that differences in the experience of robot-initiated touch might arise from societal and cultural norms (e.g., high- versus low-contact cultures [19]) and the relationship individuals have with inanimate objects (e.g. Japanese sense spiritual presence in machines and regard them as extensions of themselves [99,100]).

It remains thus open whether the findings can be replicated in other cultures or populations such as children or elderly people, user groups that have been primarily regarded in the realm of robot therapy, where touch is directed from the subjects to therapeutic robots. Nevertheless, the scenario that was used in the present study was tailored to students, and would therefore not have worked with another group of participants. However, it can be assumed that a conversational scenario with another topic that matches a broader sample would elicit comparable results.

Furthermore, the material embodiment of the robot plays a crucial role in the perception and experience of touch from a robot. Thus, the findings are so far limited to this specific kind of small-size, humanoid robot, fully covered with plastic skin. Even for the same type of robot findings might vary if the robot’s hand would be covered with a soft fabric or a heat pad to simulate a warm touch (cf. [66,67,101]). These possible influences of the robots’ material embodiment should be considered in future work.

With respect to the application of subjective measures from interpersonal communication research to human–robot interactions, low to moderate reliabilities for some (sub-)scales (i.e., Interpersonal Attraction Scale, Relational Communication Scale and IOS; see Measures) suggest that further validation of the adapted scales is necessary for a continued use to evaluate robots. However, low alpha values might as well have resulted from the small number of items per scale [102].

## Conclusions

In summary, the present study demonstrated a possible way to investigate the effects of seemingly spontaneous robot-initiated touch on human subjects without any prior warning or request. The results revealed that touch in comparison to no touch affected participants’ emotional state (assessed via self-report), in a favorable direction. Moreover, the objectively observed behavior of the participants was also affected by the touch from the robot, as manifested in more laughing and compliance in the touch condition. Due to the observed differences in the effect of the presence of touch on the affective state, as well as on the behavior during the conversation, it seems as if robot touch immediately affected the participants on a subconscious, emotional level, whereas no differences in the more cognitively effortful evaluation occurred. This can be compared to the social reactions which have been demonstrated in media equation-related research: While on a conscious level, mechanisms known from interpersonal communication are not transferred to human–robot interaction, immediate behavior is indeed affected. This lends evidence to the ethopoeia approach as formulated by [11], according to which deeply rooted social reactions are triggered by social cues shown by artificial counterparts. Still, given that pro-social behavior might not have been visible in the specific situation due to the described shortcoming (the experimenter and not the robot needed help), we nevertheless believe that the additional measurement of self-reported feelings, which demonstrate that touch from the robot had a positive effect on participants’ affect, can explain why participants were more willing to comply to the robot’s request.

In conclusion, incorporating robot-initiated touch into human–robot interaction can positively impact individuals’ experience of the interaction in conversational contexts. Furthermore, we demonstrated that touch from a robot reinforces requests and increases compliance, which could be a useful strategy if a robot is used, for example, to convince individuals to take their medication or to exercise.

However, incorporating (affective) touch into technologies and expecting the experience to be universal is too narrowly considered (see [96]). Confounding behaviors such as the robot’s co-speech are crucial variables to consider when robot touch is studied.

In addition, observed similarities in individuals’ reactions to interpersonal and robot touch bear the potential to enable more controlled investigations of interpersonal touch. By using programmed robots that look like humans (e.g., humanoid or even android), confounds such as uncontrolled nonverbal behaviors [25] can be reduced. Before this, however, more research is needed to understand what the exact differences between robot touch and human touch actually are. Therefore, the direct comparison of touch from a robot to touch from a human in an experimental setup is essential.

## Supporting information

S1 Text Verbal instruction by the experimenter before the interaction. (PDF)

S1 Dataset Data collected via questionnaire and coded behavioral observations. (XLSX)
