# Supplementary material for: The persuasive power of robot touch. Behavioral and evaluative consequences of non-functional touch from a robot
Source: PLoS One. 2021 May 5;16(5):e0249554. doi: 10.1371/journal.pone.0249554 (PMC8099132; doi:10.1371/journal.pone.0249554)
Supplement: S1 Text — (PDF) [file pone.0249554.s001.pdf]

Instruction

The experimenter said:

> In order to record your physiological data, the wristband measures your heart rate comparable to a fitness tracker by means of the silver buttons inside the wristband. In addition, your skin response is measured through your fingertips, which should be placed on the box during the conversation. Please try to place your hand on the marks on the box. The contact points are located at the upper parts of the finger marks [experimenter demonstrates how to place the hand correctly on the box]. Put your hand on the box for test purposes [experimenter checks whether the fingertips are close enough to the robot and asks for correction if necessary]. Okay thank you, the robot will tell you when to start the recording and place your hand on the box. Until then you can remove your hand from the box. I will wait outside until you have finished. Enjoy the interaction!
